# Medication Adherence in Chinese Patients with Essential Tremor: A Real World Study

**DOI:** 10.5334/tohm.1095

**Published:** 2025-09-16

**Authors:** Runcheng He, Mingqiang Li, Xun Zhou, Lanqing Liu, Chunyu Wang, Hainan Zhang, Qiying Sun

**Affiliations:** 1 Department of Neurology, the Second Xiangya Hospital, Central South University, Changsha, Hunan, China; 2 Clinical Medical Research Center for Stroke Prevention and Treatment of Hunan Province, Department of Neurology, the Second Xiangya Hospital, Central South University, Changsha, Hunan, China; 3 Department of Neurology, The First Affiliated Hospital of University of South China, Hengyang, Hunan, 421001, China; 4 Department of Geriatric Neurology, Xiangya Hospital, Central South University, Changsha, Hunan, China; 5 National Clinical Research Center for Geriatric Disorders, Xiangya Hospital, Central South University, Changsha, Hunan, China; 6 Key Laboratory of Hunan Province in Neurodegenerative Disorders, Central South University, Changsha, Hunan, China

**Keywords:** Medication adherence, Essential tremor, Essential tremor plus, Real world study

## Abstract

**Background:**

Medication adherence in essential tremor (ET) remains poorly characterized. This real world study aimed to investigate adherence rates, clinical correlates, and predictors among ET patients in China.

**Methods:**

A prospective cohort of 318 ET patients (116 pure ET, 202 ET-plus) was followed for a mean of 22.91 ± 3.86 months. Standardized assessments included the Tremor Research Group Essential Tremor Rating Assessment Scale (TETRAS), Mini-Mental State Examination (MMSE), Montreal Cognitive Assessment (MoCA), and Non-Motor Symptoms Scale (NMSS). Adherence was defined as daily use of prescribed tremor medications. Logistic regression identified predictors.

**Results:**

Only 27.4% (87/318) maintained daily adherence. ET-plus patients showed higher adherence than pure ET (32.2% vs 19.0%, P = 0.011). Arotinolol was the most common medication. Compared to non-adherent patients, adherent patients showed higher urban residency (P = 0.026), head tremor prevalence (P = 0.002), mild cognitive impairment (P = 0.038), higher TETRAS-I (P = 0.047) and TETRAS-II scores (P = 0.008), as well as lower MoCA scores (P = 0.021). Multivariable analysis showed better medication adherence was significantly associated with higher TETRAS-II score (OR = 1.041, 95% CI = 1.001–1.082, P = 0.047), urban residence (OR = 1.775, 95% CI = 1.066–2.957, P = 0.028), and the presence of head tremor (OR = 1.936, 95% CI = 1.125–3.332, P = 0.017). No significant association was found between ET subtypes and adherence (P > 0.05).

**Conclusion:**

Medication adherence is alarmingly low in Chinese ET patients, especially in pure ET. Greater tremor severity, presence of head tremor, and urban residence were independently associated with better medication adherence.

**Highlight:**

Medication adherence among Chinese essential tremor (ET) patients remains suboptimal (only 27.4% in our cohort). ET plus patients showed higher adherence (32.2%) than pure ET (19.0%). Predictors of adherence included severe tremor (TETRAS-II), urban residence, and head tremor. Arotinolol was the predominant treatment. Findings emphasize the need for personalized interventions.

## Introduction

Essential tremor (ET) is one of the most prevalent movement disorders, clinically characterized by symmetric action tremor of bilateral upper limbs, with or without tremor in other parts of the body such as the head, face, voice, or lower limbs [1]. ET affects approximately 0.9% of the global population. Epidemiological studies conducted in China have revealed significant regional variations in ET prevalence. For example, a study among individuals aged 55 and above in Beijing reported an overall ET prevalence of 3.29%, with varying rates across rural (4.29%), urban (2.85%), and mountainous (2.29%) areas [2]. However, a study conducted in a rural area of Shanghai reported a prevalence of 0.306% among residents aged 50 and older [3]. Given the obvious clinical heterogeneity of ET, the 2018 consensus statement on tremor from the International Parkinson and Movement Disorder Society proposed a new concept of ET plus, defined as ET in the presence of additional neurological signs of uncertain significance such as impaired tandem gait, questionable dystonic posturing, and mild memory impairment [4]. Nevertheless, the underlying mechanisms behind these differences remain unclear; it is ambiguous whether they signify distinct pathophysiological entities or merely represent a more advanced state along a disease continuum. Previous studies have identified shared familial aggregation and alcohol responsiveness between pure ET and ET plus, supporting the hypothesis that these conditions exist on a common disease spectrum [5,6]. Furthermore, electrophysiological evaluations have identified a similar tremor oscillator in both groups, providing additional evidence that they represent different manifestations within a spectrum of tremor disorders [7].

Despite its high prevalence, ET remains underdiagnosed and undertreated. Pharmacotherapy, including propranolol and primidone, is the first-line treatment for ET. However, long-term medication adherence rates remain suboptimal due to side effects and variable patient responses [8]. The concept of ET plus, a subtype encompassing additional neurological signs, has further complicated adherence research. ET plus patients often exhibit more severe symptoms and faster progression [5,9], which may theoretically increase adherence due to greater perceived need for treatment.

Medication adherence research holds profound implications across multiple dimensions. For patients, improved adherence enhances treatment efficacy, optimizes clinical outcomes, and ultimately elevates quality of life by ensuring consistent therapeutic benefits. At the societal level, understanding adherence patterns facilitates rational allocation of healthcare resources and informs evidence-based public health policy formulation. For healthcare systems, addressing non-adherence reduces avoidable medical expenditures, improves operational efficiency through targeted interventions, fosters multidisciplinary collaboration among clinicians, pharmacists, and behavioral specialists, and stimulates development of comprehensive adherence-enhancing strategies. Current research on medication adherence in ET remains substantially limited. Existing studies have primarily focused on Western populations [10,11], while longitudinal follow-up data from Asian cohorts are particularly scarce. Notably, no studies have systematically compared medication adherence patterns between pure ET and ET plus in the international literature. Given the progressive nature of ET and the requirement for lifelong pharmacotherapy, this significant research gap urgently needs to be addressed.

This study aims to conduct a real world study on patients with pure ET and ET plus, in order to quantify medication adherence rates in a large Chinese ET cohort, compare adherence between pure ET and ET-plus subtypes, and identify clinical predictors of adherence. Our findings provide a comprehensive analysis of medication adherence in Chinese ET patients through a longitudinal follow-up investigation, offering insights for personalized interventions and highlighting unmet needs in ET management.

## Methods

### Study Design and Participants

We conducted a real world study involving 330 patients diagnosed with ET who were undergoing pharmacotherapy, continuously recruited from the inpatients and outpatients clinic of the Department of Neurology, Xiangya Hospital of Central South University – a clinical sub-center of the National Survey of Essential Tremor Plus in China (NSETP-China) [5,9], from May 1, 2021 to April 30, 2022. This study was approved by the Medical Ethics Committee of Xiangya Hospital, Central South University and was conducted according to the principles of the Declaration of Helsinki. This study was registered at ClinicalTrials.gov (Identifier: NCT04198246). Informed consent was obtained from each patient. All patients were diagnosed by at least two experienced neurologists according to the 2018 International Movement Disorder Society Tremor Group essential tremor diagnostic criteria [4]. We defined ET plus using the new consensus criteria, i.e., ET patients with any of the following features: mild cognitive impairment (defined as MMSE total score illiterate < 17, elementary education < 20, middle school education or above < 24, or MoCA total score < 26, the standard scoring adds a 1 point correction for participants with 12 or fewer years of education), questionable dystonic posturing (defined as UDRS score ≥ 1), impaired tandem gait (defined as at least two missteps out of a 10-step trial), rest tremor (UPDRS-III score for rest tremor ≥1), and questionable myotonia (established by neurologic examination). Patients undergoing treatment for tremor were evaluated following the cessation of medication. Patients were evaluated two times: at baseline, and again at approximately 2 years after baseline. The follow-up evaluations were performed by neurologists using face-to-face methods such as hospital visits, community clinic consultations, and home surveys, as well as telephone interviews.

### Clinical evaluation

Demographic and clinical data were collected into the Parkinson’s Disease and Movement Disorders Multicenter Database and Collaborative Network in China (PD-MDCNC, http://pd-mdcnc.com). All patients underwent neurological examination and neuropsychological assessment at baseline and follow-up.

Tremor was evaluated with the Tremor Research Group Essential Tremor Rating Assessment Scale (TETRAS) [12]. TETRAS-I was used to evaluate the impact of tremors on daily living, while TETRAS-II was used to evaluate tremor distributions and severity. Global cognitive function was assessed using the Mini-Mental State Examination (MMSE) [13] and Montreal Cognitive Assessment (MoCA) [14]. Dystonic posturing was evaluated with the Unified Dystonia Rating Scale (UDRS). The Non-Motor Symptoms Scale (NMSS) evaluate the severity of non-motor symptoms. The Scale for the Assessment and Rating of Ataxia was used to assess ataxia severity. Tandem gait was assessed by asking patients to take 10 consecutive tandem steps along a straight line while keeping the arms at the sides. Patients were allowed to complete 2 attempts serially, missteps were recorded in every turn, and the best performance was recorded. The patients were assessed for rest tremor, suspected rigidity, and the severity of Parkinson’s disease (PD) using the Unified-Parkinson Disease Rating Scale (UPDRS). The Rapid-eye-movement sleep behavior disorder questionnaire-Hong Kong (RBDQ-HK) [15].

### Assessment of medication

Regarding medication usage, patients were queried about the following: (1) whether they were currently taking any medication to alleviate their tremor symptoms, and (2) the precise classification, dosage, and duration of the medication usage. Medication adherence was defined as not missing any medication in the past 3 months.

### Statistical analysis

Continuous variables following normal distribution were expressed as mean±standard deviation, whereas non-normally distributed data were described using the median (25th percentile, 75th percentile). The results for categorical variables are presented as percentages and frequencies. To compare the distribution of continuous variables between different groups, the nonparametric Mann–Whitney U test was used. Binary variables were compared using the chi-square test. Binary logistic regression model was used to investigate factors associated with adherence to anti-tremor medication. Variables that showed statistical significance (P < 0.05) in univariate analyses were included in a binary logistic regression model using the Enter method. Significance was considered if P < 0.05. Statistical analysis was performed using IBM SPSS version 22.0.

## Results

### Overview

In the real world study, 12 ET patients were lost to follow-up due to refusal or incorrect contact information. 286 ET patients completed face-to-face follow-up. 32 ET patients completed only telephone follow-up due to a lack of cooperation for face-to-face visits. We focused on investigating medication adherence in 318 ET patients, excluding 12 patients who were lost to follow-up.

Among the 318 study participants, there were 116 (36.5%) pure ET patients with an average age of 54.46 ± 15.17 years, an average disease duration of 10.22 ± 8.98 years, and an average age at onset of 44.24 ± 17.17 years, including 69 male patients (59.5%). There were 202 (63.5%) ET plus patients with an average age of 61.11 ± 13.82 years, an average disease duration of 13.25 ± 11.38 years, and an average age at onset of 48.69 ± 16.48 years, including 85 male patients (42.1%). The average follow-up interval was 22.91 ± 3.86 months.

### Medication adherence in ET

**Figure 1:**
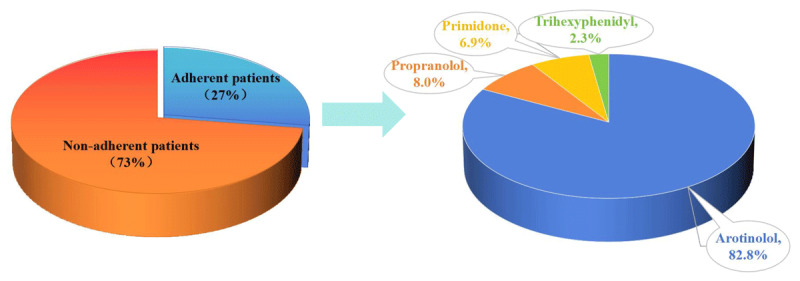
Medication Status of Patients with Essential Tremor.

87 ET patients were adherent to anti-tremor medications with a prevalence of 27.4%. ET plus patients had a higher odds of being adherent to medication compared to pure ET patients (32.2% vs 19.0%, χ^2^ = 6.473, P = 0.011). As concerns the anti-tremor medications, 72 (22.6%) patients used arotinolol, 7 patients (2.2%) took propranolol, 6 patients (1.9%) took primidone, and 2 patients (0.6%) took trihexyphenidyl (shown in Figure 1). Among 231 ET patients with non-adherent medication, 97 patients (42.0%) discontinued treatment due to perceived inefficacy, 67 patients (29.0%) declined pharmacotherapy as they considered their symptoms mild and not significantly affecting daily life, 39 patients (16.9%) were influenced by other factors including economic constraints and adverse drug effects, and 28 patients (12.1%) reported difficulties in accessing medications due to transportation issues or residing in remote areas.

In comparison to patients with non-adherent medication, those with medication adherence were more likely to reside in urban areas (58.6% vs 44.6%, χ^2^ = 4.982, P = 0.026) and had a higher prevalence of head tremor (42.5% vs 25.1%, χ^2^ = 9.155, P = 0.002). Additionally, Mild cognitive impairment (40.2% vs 28.1%, χ^2^ = 4.286, P = 0.038) was also significantly more prevalent in patients with adherent medication. Moreover, patients with adherent medication had higher scores in TETRAS-I (13 (8, 20) vs 15 (10, 22.5), Z = –1.990, P = 0.047), and TETRAS-II (17 (14, 21) vs 20 (15, 24.75), Z = –2.658, P = 0.008), as well as lower score in MoCA (26 (23, 27.5) vs 25 (21, 27), Z = –2.303, P = 0.021) than patients with non-adherent medication (Table 1).

**Table 1:** Comparison of demographic and clinical characteristics between the adherent and non-adherent patients to medication.


VARIABLES	NON-ADHERENT (n = 231)	ADHERENT (n = 87)	*P* VALUE

Gender (male, %)	113 (48.9%)	41 (47.1%)	0.776

Age (years)	62 (51.5, 69)	62 (54.5, 68)	0.646

Age at onset (years)	48 (35, 60)	52 (42.5, 60)	0.483

Duration (years)	10 (4, 20)	8 (4, 12)	0.239

Education level (years)	11 (6, 12)	9 (6, 12)	0.396

Area (urban/rural)	103/128	51/36	**0.026**

Tremor distribution			

Head tremor (%)	58 (25.1%)	37 (42.5%)	**0.002**

Face tremor (%)	62 (26.8%)	25 (28.7%)	0.735

Voice tremor (%)	63 (27.3%)	27 (31.0%)	0.507

Lower limbs tremor (%)	57 (24.7%)	23 (26.4%)	0.747

ET plus (%)	137 (59.3%)	65 (74.7%)	**0.011**

Mild cognitive impairment	65 (28.1%)	35 (40.2%)	**0.038**

Rest tremor	34 (14.7%)	13 (14.9%)	0.960

Questionable dystonic posturing	50 (21.6%)	25 (28.7%)	0.184

Impaired tandem gait	42 (18.2%)	20 (23.0%)	0.335

TETRAS-I score	13 (8, 20)	15 (10, 22.5)	**0.047**

TETRAS-II score	17 (14, 21)	20 (15, 24.75)	**0.008**

MMSE score	28 (25, 29)	27 (24, 29)	0.094

MoCA score	26 (23, 27.5)	25 (21, 27)	**0.021**

NMSS score	11 (4, 20)	12 (6, 19)	0.607

Cardiovascular subscore	0 (0, 0)	0 (0, 0)	**0.023**

Sleep/fatigue subscore	3 (0, 6)	3 (0, 6)	0.541

Mood/cognition subscore	0 (0, 2)	0 (0, 3)	0.208

Perceptual problems subscore	0 (0, 0)	0 (0, 0)	0.302

Attention/memory subscore	3 (0, 4)	4 (0, 4)	0.140

Gastrointestinal subscore	0 (0, 1)	0 (0, 2)	0.255

Urinary subscore	0 (0, 2)	0 (0, 2)	0.456

Sexual function subscore	0 (0, 0)	0 (0, 0)	0.457

Miscellaneous subscore	0 (0, 2)	0 (0, 2)	0.927


Note: TETRAS = Tremor Research Group Essential Tremor Rating Assessment Scale; MMSE, Mini-Mental State Examination; MoCA, Montreal Cognitive Assessment; NMSS = Non-motor Symptoms Scale. Bold values represent statistically significant differences.

After adjusting for potential confounders in multivariable logistic regression, higher TETRAS-II scores (OR = 1.041, 95% CI = 1.001–1.082, P = 0.047), urban residence (OR = 1.775, 95% CI = 1.066–2.957, P = 0.028), and head tremor (OR = 1.936, 95% CI = 1.125–3.332, P = 0.017) were independently associated with improved adherence to anti-tremor medication (Table 2).

**Table 2:** Factors associated with adherence to anti-tremor medication.


INDEPENDENT VARIABLES	UNIVARIATE *P*–VALUE*	ODDS RATIO/95% CI	MULTIVARIATE *P*–VALUE

**GENDER (MALE, %)**	0.776	–	Not included

Age (years)	0.976	–	Not included

Age at onset (years)	0.303	–	Not included

Duration (years)	0.120	–	Not included

Education level (years)	0.979	–	Not included

Area (urban)	**0.025**	1.775/1.066–2.957	**0.028**

Head tremor	**0.003**	1.936/1.125–3.332	**0.017**

Face tremor	0.768	–	Not included

Voice tremor	0.507	–	Not included

Lower limbs tremor	0.747	–	Not included

Mild cognitive impairment	**0.039**	0.720/0.330–1.571	0.106

Rest tremor	0.960	–	Not included

Questionable dystonic posturing	0.186	–	Not included

Impaired tandem gait	0.336	–	Not included

TETRAS–I score	0.089		Not included

TETRAS–II score	**0.007**	1.041/1.001–1.082	**0.047**

MMSE score	0.125	–	Not included

MoCA score	**0.048**	0.978/0.891–1.074	0.144

NMSS score	0.792	–	Not included


Note: TETRAS = Tremor Research Group Essential Tremor Rating Assessment Scale = MMSE, Mini-Mental State Examination; MoCA = Montreal Cognitive Assessment; NMSS = Non-motor Symptoms Scale. Bold values represent statistically significant differences.

## Discussion

In this real world study, we showed that medication adherence among ET patients is suboptimal. We reported a prevalence of adherence to anti-tremor medications to be 27.4%, which is lower than foreign studies [8,10,16,17]. This discrepancy may be partially explained by our extended follow-up duration of approximately 24 months, as sustained pharmacological treatment has been shown to progressively diminish medication adherence over time. Most ET patients exhibit poor adherence to medication, which may be attributed to limited effectiveness of these medications and the emergence of drug resistance [18,19]. The leading causes of non-adherence in our study were perceived drug inefficacy, tolerable symptoms, access limitations, economic constraints, and adverse effect concerns. Furthermore, the available options for medications are relatively limited, and no new effective drugs have been developed in recent years.

The pharmacological management patterns observed in our Chinese ET cohort reveal several noteworthy findings regarding first-line treatment preferences. Arotinolol, propranolol, and primidone emerged as the most frequently prescribed medications, consistent with their status as first-line agents in Chinese treatment guidelines. However, the particularly high prescription rate of arotinolol (82.8% of adherent ET patients) warrants special consideration. This pattern stands in marked contrast to Western cohorts, where propranolol and primidone typically dominate first-line therapy [8,11,20].

Several factors may explain the predominance of arotinolol in our cohort. A key reason is its superior tolerability compared to primidone. Although primidone is often effective, its use is frequently limited by a high incidence of dose-limiting adverse effects such as acute sedation, dizziness, and nausea. In contrast, β-blockers was associated with a more favorable side effect profile, suggesting that it may represent a more sustainable treatment option for a broader ET patient population. Beyond tolerability, drug accessibility serves as another critical factor influencing real-world treatment decisions. β-blockers are more readily accessible within the Chinese healthcare system than primidone. A notable advantage of arotinolol, in particular, lies in its dual blockade of α1- and β-adrenoceptors. This distinctive pharmacological characteristic enables partial mutual neutralization of adverse effects typically associated with selective receptor blockade. For example, its α1-blocking activity may counteract the peripheral vasoconstriction and heart rate reduction induced by beta-blockers, while also mitigating adverse effects on glucose and lipid metabolism. In addition, arotinolol demonstrates limited permeability across the blood-brain barrier, a characteristic that differentiates it from propranolol. Consequently, central nervous system related adverse events—such as dizziness, fatigue, and mental depression—occur significantly less frequently [21,22]. Given these considerations, arotinolol has become the most commonly used medication for ET in China. Future large-scale, prospective studies are warranted to directly compare the long-term efficacy and adherence rates between these agents.

It is also noteworthy that while our cohort included a substantial number of patients with head tremor, the severity was generally mild and did not necessitate treatment with botulinum toxin injections or any other non-oral treatments (such as deep brain stimulation or magnetic resonance image guided focused ultrasound). Following our center’s clinical protocol, botulinum toxin therapy is typically reserved for patients with more severe, disabling, or medication-resistant head tremor. Since the patients in our study population predominantly had mild symptoms and were seeking first-line oral pharmacological treatment, botulinum toxin was not utilized.

Our study further highlights significant variations in adherence between ET subtypes, with ET plus patients demonstrating higher adherence (32.2%) compared to pure ET patients (19.0%). This discrepancy may be attributed to the more severe and multifaceted symptomatology of ET plus, which includes mild memory impairment, impaired tandem gait, and other neurological signs, thereby increasing patients’ perceived need for treatment. However, the high prevalence of mild cognitive impairment in ET plus patients may have a dual—and paradoxical—effect on adherence. On one hand, cognitive deficits can increase dependence on caregivers for medication management; on the other hand, they may compromise the patient’s own ability to adhere to treatment regimens [23]. Patients with mild cognitive impairment may be more likely to persist with treatment due to heavier disease burden or more consistent caregiver involvement. Indeed, patients with more pronounced cognitive decline often receive greater assistance and supervision with medications, which may contribute to higher adherence rates. Conversely, pure ET patients, who typically exhibit fewer motor and non-motor symptoms, may perceive their tremor as less debilitating, leading to lower adherence.

We revealed a significant relationship between tremor severity and medication adherence, with each 1-point increase in TETRAS-II scores associated with a 4.1% increase in the odds of medication adherence. This relationship suggests that patients with severe tremor symptoms are more likely to recognize the necessity of treatment due to significant impacts on daily living activities, whereas those with milder symptoms may underestimate the need for medication as their condition causes minimal disruption to daily functioning. This aligns with previous research demonstrating that disease severity is a key determinant of adherence in chronic neurological conditions. We further identified head tremor and urban residence as two critical determinants of medication adherence in ET patients, which may be mechanistically explained by the following observations. As a highly visible motor manifestation, head tremor may significantly affect patients’ physical appearance and social presentation. This “visible” symptom likely induces psychological distress (e.g., social anxiety or self-image concerns), thereby motivating patients to maintain medication adherence more rigorously to mitigate its cosmetic impact. Urban dwellers may benefit from structural advantages in healthcare accessibility, including: greater density of medical facilities reducing travel burden for prescription refills, and more robust health insurance coverage and medication supply systems. These systemic factors may collectively promote long-term treatment persistence among urban patients.

Our study did not identify an independent association between ET Plus and medication adherence, supporting the hypothesis that ET plus and pure ET may not represent distinct clinicopathological entities. This finding is consistent with recent comparative studies on thalamic deep brain stimulation, which have reported similar tremor-suppressing effects in patients with ET and those with ET plus, indicating comparable therapeutic efficacy between the two groups [24,25,26]. These outcomes align with the disease staging theory proposed by Lenka and Louis [27], which posits that ET plus may correspond to a specific stage within the spectrum of disease progression.

This study provides critical insights into medication adherence among Chinese ET patients, revealing that patients were more likely to adhere to anti-tremor medication if they had a higher TETRAS-II score, resided in urban areas, or exhibited head tremor. The higher adherence in ET plus patients underscores the need for subtype-specific management strategies, while the predominance of arotinolol highlights regional disparities in treatment practices. In future, the development of novel therapies could significantly improve adherence and outcomes for ET patients.

This study possesses several notable strengths. Its prospective design and extended longitudinal follow-up period minimize recall bias and allow for a more robust assessment of clinical progression over time. Furthermore, our rigorous approach to differentiating classic ET from ET plus phenotypes enhances the diagnostic accuracy and provides valuable insights into the potential heterogeneity of the disorder. Despite these strengths, several limitations must be acknowledged. First, the single-center nature of our study may limit the generalizability of our findings. The participant population was recruited from a tertiary referral center, which may not be fully representative of the broader community-based ET population, potentially introducing selection bias. Second, medication adherence was assessed solely through self-report. The incorporation of objective measures, such as electronic pill monitors or drug level assays, in future studies would provide a more reliable assessment. Additionally, although we employed standardized rating scales, the assessment of tremor characteristics remains subject to a degree of inherent subjectivity. Future research should aim to replicate these findings in larger, multi-center cohorts to enhance external validity. Longitudinal studies incorporating objective adherence monitoring and advanced neuroimaging biomarkers are also warranted to further elucidate the underlying mechanisms of disease progression and treatment response in ET.

## Conclusions

Medication adherence among Chinese ET patients remains suboptimal. Pure ET patients demonstrated markedly poorer adherence compared to ET plus patients. Disease severity, urban residence, and the presence of head tremor were identified as significant predictors of medication adherence in ET patients. Our study highlights the urgent need to develop personalized medication adherence intervention strategies.
